# The feasibility, acceptability and outcomes of exergaming among individuals with cancer: a systematic review

**DOI:** 10.1186/s12885-018-5068-0

**Published:** 2018-11-21

**Authors:** Daniel Tough, Jonathan Robinson, Steven Gowling, Peter Raby, John Dixon, Samantha L. Harrison

**Affiliations:** 1 School of Health and Social Care, Teesside University, Middlesbrough, TS1 3BX UK; 2 Department of Sport and Exercise Sciences, University of Sunderland, Sunderland, SR1 3SD UK

**Keywords:** Cancer, Exergaming, Active video games, Rehabilitation

## Abstract

**Background:**

Individuals with cancer have reduced quality of life, functionality, range of motion, strength, and an increase in pain and fatigue. Exergaming appears to be an effective rehabilitation tool for Parkinson’s disease, multiple sclerosis and post-stroke patients to improve functionality, balance and quality of life; however, the usefulness of exergaming in individuals with cancer is unknown. The aim of this systematic review is to describe exergaming interventions delivered to adults with a current or previous cancer diagnosis and to report the feasibility, acceptability and outcomes of such interventions.

**Methods:**

Studies reporting on exergaming interventions delivered to individuals with a current or previous cancer diagnosis were included. 12 electronic databases were searched. Eight articles (seven interventions) were identified. Data were extracted and assessed for quality by two reviewers.

**Results:**

Three interventions were delivered at hospital, two at home, one at a clinical laboratory, and one did not report. Two interventions were delivered by a physiotherapist, two by an occupational therapist, and one by a nurse, research staff and an exercise physiologist. The Nintendo Wii was used in four of seven studies, whilst the remaining three used the IREX system, BrightArm Duo Rehabilitation System or a custom made exergame. Studies showed that most participants enjoyed the exergaming intervention, and would recommend their use, with some preferring exergaming over standard care interventions. Adherence rates and enjoyment appear greater during exergaming than standard care. Exergaming interventions appear to support improvements balance, function, physical activity levels, strength, fatigue, emotions, cognition and pain.

**Conclusion:**

Exergaming interventions delivered to individuals with cancer show great heterogeneity; differing in duration, frequency and gaming platform. The disease stage and severity of those included, and the outcome measures assessed also vary widely making it difficult to conclude its effectiveness at this time. However, adherence rates and enjoyment appear greater during exergaming compared to standard care, supporting the feasibility and acceptability of this type of intervention delivery for adults with cancer.

## Background

Cancer is the second leading cause of deaths worldwide, with 8.8 million cancer-related deaths in 2015 alone [1, 2]. Cancer patients commonly experience symptoms such as cancer-related fatigue (CRF), decreased functionality and range of motion (ROM), decreased strength, pain, insomnia and mood disturbances, leading to a decrease in quality of life (QOL), following surgery or pharmaceutical treatments [3–7]. The American College of Sports Medicine recommend that individuals with cancer should avoid inactivity and return to normal daily activities as soon as possible if they undergo surgery, or continue daily routines and exercise if undergoing non-surgical treatments [8]. The National Comprehensive Cancer Network [9] supports this and recommends starting rehabilitation upon diagnosis, continuing up to, and following, completion of treatment.

A lack of physical activity has been associated with an increase in mortality rates [10–12] and a higher disease recurrence [13]. Previous systematic reviews indicate that exercise interventions, consisting of aerobic and resistance training, delivered to individuals with a diagnosis of cancer significantly alleviates some side-effects of surgery, including CRF, and improves exercise capacity and QOL [14–17]. Adherence to such exercise interventions is poor (< 50% of prescribed sessions attended on occasions) [16, 18], potentially due to exercises being monotonous [19]. Current rehabilitation strategies for those with cancer advocate exercises such as stretching, ROM exercises, fine motor training, yoga, aerobic and resistance training exercises [7, 20]. In one study, supervised exercise sessions had an adherence rate of 73%, compared to only 8.7% in a home-based program [21]. Adherence to an exergaming intervention has shown to be greater than among those undergoing pulmonary rehabilitation [22] and dementia [23].

Exergaming, defined as the combination of exercise and gaming, is a relatively new intervention idea, whereby the user must use physical movements in order to interact with a game [24]. Such games can be played through hand-held controllers (Nintendo Wii), physical movement captured through video-cameras (Sony EyeToy and Microsoft Xbox Kinect) or weight-sensing platforms (Dance Dance Revolution [DDR] and Nintendo Wii Fit) [25]. Exergaming has been found to be an acceptable method for exercising among older adults [26, 27]. It has also been found to be safe [28–30], easy to use [26] and enjoyable [26, 27]. Exergaming consoles are relatively inexpensive, with the Nintendo Wii and Microsoft Xbox Kinect currently costing less than £250 and £300, respectively. Recent systematic reviews have demonstrated the feasibility, acceptability and effectiveness of exergaming at improving balance, functionality, cognition and QOL in individuals with chronic conditions, including Parkinson’s disease (PD) [24], multiple sclerosis (MS) [31], cystic fibrosis [32] and those who have recently suffered a stroke [33]. Exergaming has also been found to be a fun and enjoyable method of physical activity, potentially increasing one’s motivation to partake in exercise programs [34]. The benefits of exergaming in other clinical populations are becoming increasingly recognised, however there is limited research assessing the feasibility, acceptability and effectiveness of exergaming interventions amongst patients with a current or previous cancer diagnosis.

To our knowledge, no systematic review has synthesised the evidence of exergaming among adults with a current or previous cancer diagnosis. It is therefore important to review the current literature to examine whether an exergaming intervention can indeed support cancer rehabilitation. It will also help to identify the best mode of intervention delivery for this clinical population, guiding future studies. The aim of this systematic review is three-fold; 1) To describe exergaming interventions applied in individuals with cancer, 2) To assess the feasibility and acceptability of an exergaming intervention delivered to adults (18+ years) with a current or previous cancer diagnosis, and 3) To explore the outcomes of such an intervention in this clinical population. Gathering such evidence in a systematic way may help to inform early exergaming interventions for individuals with cancer, within a hospital or home environment, as an alternative to standard mobilisation and exercise therapy.

## Methods

The systematic review is registered with PROSPERO: CRD42017054615. The reporting of the review is consistent with PRISMA guidelines [35].

### Search strategy

The search strategy was developed by one reviewer (DT) in conjunction with a professional librarian. The titles, keywords and abstracts of each article, where applicable, were searched with set search terms (Appendix 1). MeSH headings were searched for ‘exergaming’ and ‘cancer’, or the nearest available terms, to identify relevant search terms. The references of the included articles were also checked.

The following electronic databases were searched: CINAHL, MEDLINE, AMED, PEDro, Cochrane Library Online, ScienceDirect, SPORTDiscus, EMBASE, ASSIA, Scopus, Nursing and Allied Health Source and PsycINFO. All databases were searched from inception to January 2017.

### Screening process

The main researcher (DT) checked for, and removed, all duplicated publications from the initial search. All titles and abstracts were screened independently by two reviewers (DT and SG/PR). Any publications which the reviewers were in disagreement over were resolved through a discussion until a consensus was reached. Upon identifying relevant articles, all reviewers evaluated the full text of these articles against the study’s inclusion criteria.

### Article selection

Participants, intervention, comparison, outcomes and study design were used to identify the inclusion and exclusion criteria for the study.

#### Participants

Adults (≥18 years) with a current or previous diagnosis of cancer at the time of testing, regardless of whether surgery had been performed.

#### Intervention

Studies reporting on interventions involving exergames as part of the protocol were included. The exergames must have encouraged exercise and physical activity, in order to interact with the game. Any studies commenting on feasibility and acceptability of such interventions were included.

#### Comparison

No comparative groups were required to be included.

#### Outcomes

All health outcomes were considered within the review including, but not limited to, CRF, QOL, balance, fitness, strength and ROM. Any feasibility and acceptability findings, including adherence rates, were included.

#### Study design

Inclusion was not limited by trial design. Studies using both qualitative and quantitative methods were included within the review. Any reviews of the literature were excluded from the study. All articles were required to be available in English, as well as being published within peer-reviewed journals.

### Data extraction

Data extraction was carried out by one reviewer (DT) before being verified by a second reviewer (SG). Participant characteristics, the disease, equipment used, intervention details such as the frequency, duration and setting, comparison groups, outcome measures and significant findings were all extracted from the chosen articles.

### Study quality assessment

The Cochrane Collaboration’s tool was used to assess the quality of any controlled trials [36]. This tool uses seven bias domains to enable a user to judge each aspect as a high, low or unclear risk of bias, allowing readers to critically assess the quality of an intervention [37]. For observational studies, the Quality Checklist for Healthcare Intervention Studies was used [38]. This checklist, consisting of 27 items, has been described as one of the best evaluation checklists available due to its ease of use, high reliability and validity and suitable for systematic reviews [39]. It provides an overall study quality index and includes categories addressing reporting, external quality, internal validity-bias, internal validity-confounding and a power rating [40]. The Critical Appraisal Skills Program (CASP) tool [41] was used for any qualitative studies. The qualitative CASP checklist helps users to review an article based upon 10 questions that rate the credibility, relevance and rigour of the study [42]. It allows for easy identification of what a study is lacking, whilst being able to compare study qualities to one another [43]. Each identified article was assessed for risk of bias with the relevant aforementioned tool by two reviewers (DT and SG) independently, before conferring, with any disagreements resolved through a discussion.

### Data analysis

Upon extracting all appropriate data from relevant journal articles, a descriptive summary was conducted to describe the interventions, assess the feasibility and acceptability of exergaming. The outcomes of the interventions were also described among patients with a current or former diagnosis of cancer.

## Results

**Fig. 1:**
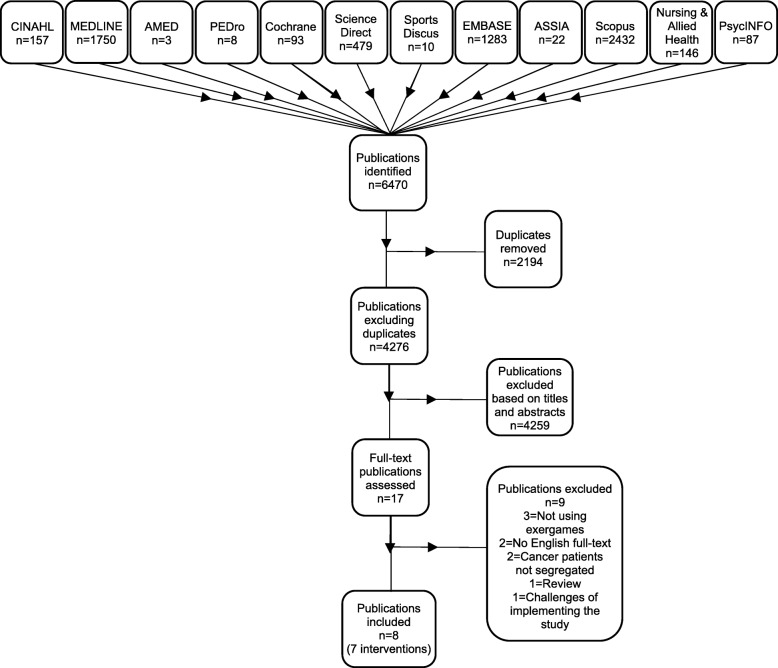
PRISMA Flow diagram of the literature search strategy used

A total of 4276 articles were screened (excluding duplicates). 17 full text articles were identified and reviewed. Nine studies were excluded from the review; three didn’t involve exergaming, two weren’t available in English, in two articles the cancer patients weren’t segregated from the other participants, one was a review of the literature which wasn’t cancer specific and also didn’t involve exergaming and the final article didn’t explore the feasibility, acceptability or outcomes of an exergaming intervention (Fig. 1). Of the eight remaining studies, one intervention [44] was a continuation of a previous intervention [45] by the same authors the previous year; therefore, these two were combined so as not to allocate unfair weighting to this study.

### Study quality design and assessment

One randomised controlled trial [46] and one controlled trial were included [20], both of which were assessed for risk of bias using the Cochrane Collaboration’s tool. The quality assessment didn’t detect a high risk of bias for either study (Table 1).

**Table 1:** Study quality assessment for controlled trials using the Cochrane Collaboration’s Tool

Author (Date)	Selection bias	Allocation Concealment	Performance bias	Detection bias	Attrition bias	Reporting bias	Other
Sajid, et al. [46]	*Low* - Participants were randomised into one of three groups	*Unclear* - Randomisation concealment not specified	*Low* - Blinding of participants not possible. Investigators blinded	*Low* - Investigators, study statistician and data managers were blinded throughout.	*Unclear* - Not all outcome data was reported (6MWT)	*Unclear* - No reporting of one variable (6MWT)	Details regarding Wii intervention are vague and lack detail
Yoon, et al. [20]	*Unclear* - Stratified random sampling used	*Unclear* - Concealment unspecified	*Unclear* - Blinding of participants not specified	*Low* - Evaluation performed by a blinded occupation therapist	*Low* - No missing outcome data.	*Low* - All outcomes reported and discussed sufficiently.	

One study [26] was an exploratory study which was assessed using the CASP tool. Most of the aspects were considered by the authors, with only four from 10 rated as ‘Can’t tell’ (Table 2).

**Table 2:** Study quality assessment for qualitative studies [26] using the CASP Tool

	Yes	No	Can’t tell	Comments
1) Was there a clear statement of the aims of the research?	✓			Aim and rationale of the study is clear.
2) Is a qualitative methodology appropriate?	✓			Methodology necessary in order to evaluate participants’ experiences.
3) Was the research design appropriate to address the aims of the research?	✓			Research design explained and justified in accordance with research goals.
4) Was the recruitment strategy appropriate to the aims of the research?	✓			Clear explanation as to how participants were recruited and justified sample size.
5) Was the data collected in a way that addressed the research issue?			✓	Data collection setting and data collection method wasn’t justified. Detail as to how interviews were recorded and transcribed.
6) Has the relationship between researcher and participants been adequately considered?	✓			Researcher informs of no relationship with the participants.
7) Have ethical issues been taken into consideration?			✓	Brief explanation of how participants were informed of the research and no detail on maintenance of ethical standards.
8) Was the data analysis sufficiently rigorous?			✓	Brief detail of data analysis. Semi-structured interview could be source of bias.
9) Is there a clear statement of findings?			✓	Research hasn’t discussed credibility of their findings. No information regarding second analyst for transcriptions and interpretations.
10) How valuable is the research?	✓			Research contributes to existing knowledge and identifies new areas to be researched

The remaining four studies were assessed using the Quality Checklist for Healthcare Intervention Studies (Table 3). Of these four studies, three were feasibility interventions [44, 45, 47, 48], with the final study a single-subject case study [49]. The three feasibility studies showed relatively good study quality with the lowest score being 20/30, whilst the case study was rated relatively low at 11/30 [49].

**Table 3:** Study quality assessment for observational studies using the Quality Checklist for Healthcare Intervention Studies [35]

Author (Date)	Q1	Q2	Q3	Q4	Q5	Q6	Q7	Q8	Q9	Q10	Q11	Q12	Q13	Q14	Q15	Q16	Q17	Q18	Q19	Q20	Q21	Q22	Q23	Q24	Q25	Q26	Q27	sum
Betker, et al. [49]	Y	Y	N	Y	N	Y	N	N	Y	N	UD	UD	Y	UD	N	Y	Y	Y	UD	Y	UD	UD	UD	UD	N	UD	B	11
Hoffman, et al. [44, 45]	Y	Y	Y	Y	N	Y	Y	Y	Y	N	Y	Y	Y	N	N	Y	Y	Y	Y	Y	Y	N	N	N	N	Y	E	22
House, et al. [47]	Y	Y	Y	Y	N	Y	Y	Y	Y	Y	Y	Y	N	N	N	Y	N	Y	Y	Y	Y	UD	N	N	N	Y	D	20
Tsuda, et al. [48]	Y	Y	Y	Y	P	Y	Y	Y	Y	Y	UD	UD	Y	N	N	Y	N	Y	N	Y	Y	N	N	N	N	Y	F	21

*Y* Yes, *N* No, *UD* Unable to determine, *P* Partially, *B* Size of smallest intervention group is 1–2, D = Size of smallest intervention group is 5–6, E = Size of smallest intervention group is 7–8, F=Size of smallest intervention group is 8+

### Study populations

The average number of participants with cancer who completed the studies was 12 ± 12, ranging from one, in the case-study [49], to 40. The average age of the participants was 57 ± 17 years, with a range of 20–75 years, with both males (55%) and females (45%) included. The diagnosis of participants included breast, lung, brain, rectal, oesophageal, tongue and prostate cancer, as well as leukemia and lymphoma. The severity and stage of cancer varied. The sole participant in the study from Betker, et al. [49] had a cerebella tumour with severe ataxia, whilst those who partook in the intervention by Hoffman, et al. [44, 45] all had lung cancer, ranging from stage 1A to 3A (Table 4).

**Table 4:** Participant characteristics and study details of included studies

Author (Date)	Study Design	Population: n enrolled, n completed/relevant (gender), mean age (range), location	Disease (Severity/Stage)	Timing of Intervention Delivery	Setting
Betker, et al. [49]	Single subject case-study	3, 1 (male), 20 (NS), Canada	Cerebellar tumour (Severe ataxia)	NS	Hospital
Hoffman, et al. [44, 45]	Feasibility study	9, 7 (2 male), 64.6 (53–73), USA	Lung cancer (Stage 1A = 1, 1B = 1, 2A = 1, 2B = 2, 3A = 2)	Pre-surgery, post-surgery, weekly follow-ups for 16 weeks.	Home
House, et al. [47]	Feasibility study	12, 6 (0 male), 57.8 (22–78), USA	Breast cancer (NS)	9.5 years post-surgery	Clinical Laboratory
Jahn, et al. [26]	Exploratory study with qualitative evaluation.	11, 7 (5 male), 56.6 (47–70), Germany	1 = Rectal cancer, 1 = Brain metastases & breast cancer, 1 = Oesophageal cancer, 1 = Tongue cancer, 3 = Lung cancer (NS)	Receiving treatment for current cancer diagnosis	Hospital
Sajid, et al. [46]	Randomised three-arm pilot study	31, 19 up to 6 weeks (19 male), 13 after 6 weeks. Wii Grou*p =* 8 (n-3 after 6 weeks), 77.5 (70–87). EXCAP grou*p =* 6 (n-1 after 6 weeks), 75.7 (67–93). Usual Care = 5 (n-2 after 6 weeks), 71.8 (67–80). USA	Prostate cancer (NS)	62 month after initial ADT	Home
Tsuda, et al. [48]	Feasibility study	16, 9 (6 male), 67.4 (61–76), Japan	6 = Leukaemia, 3 = Lymphoma (NS)	Receiving treatment for current cancer diagnosis	Hospital
Yoon, et al. [20]	Controlled study	47, 40 (17 male). IREX grou*p =* 20 (9 male), 48.6 (NS). COT = 20 (8 male), 50 (NS). South Korea	Brain tumour (NS)	9 month after diagnosis	NS

*NS* Not specified, *EXCAP* Home-based walking and resistance intervention, *ADT* Androgen deprivation therapy, *IREX* Interactive Rehabilitation and Exercise, *COT* Conventional occupational therapy

### Study interventions

Four of the included interventions implemented the Nintendo Wii for their exergaming intervention [26, 44–46, 48]. The remaining studies used a custom made centre of pressure (COP) video game combined with a pressure mat for interaction [49], a BrightArm Duo Rehabilitation System [47] or the Interactive Rehabilitation and Exercise (IREX) system [20]. Session duration ranged from 20 to 50 min; however, not all durations were reported, as this was sometimes determined by the patients. Frequency of the sessions ranged from two days per week to at least five days per week. Again this was unclear as to the maximum frequency the intervention was carried out as it depended upon each individual’s confidence and motivation to persist. The duration of the interventions was on average 6.3 weeks, ranging from five days to 16 weeks. Only two of the studies included a comparison group [20, 46]. The study by Sajid, et al. [46] included an exergaming group (Wii), an exercise group (EXCAP) and a usual care group. The study by Yoon, et al. [20] included an exergaming group (IREX) combined with conventional occupational therapy (COT) and a COT only group (Table 5).

**Table 5:** Intervention details of included studies

Author (Date)	System	Game(s)	Intervention	Comparison Group(s)	Delivered by	Outcome Measures (Method of assessment (Significant findings))
Betker, et al. [49]	COP-controlled video game-based exercise tool with pressure mat.	Under Pressure, Memory Match and Tic-Tac-Toe.	3 sessions/week for first two weeks, 2 sessions/week during third week. 45 min per session. 3 week duration.	None	Physiotherapist	*Feasibility/Acceptability*: (Custom questionnaire) *Balance*: Fall count, COP excursion limits and COP sway path (Pressure mat). *Emotions/Cognition*: (Custom questionnaire).
Hoffman, et al. [44, 45]	Nintendo Wii	Wii Fit Plus: Downhill skiing, soccer, golf and video game activities.	Walking: 5 sessions/week, 5 min per session during week one, increase by 5 min per day each week if perceived self-efficacy > 70%. Balance: 5 sessions/week. 16 week duration.	None	Nurse	*Feasibility/Acceptability*: Acceptability and adherence (Recruitment rate, adherence %, custom questionnaire). *Balance*: Perceived balance self-efficacy (Activities-Specific Balance Confidence Scale) *Fatigue*: Cancer-related fatigue severity (BFI), perceived fatigue self-management self-efficacy (PSEFSM). *Physical Activity*: Perceived walking duration self-efficacy (Self-efficacy for Walking Duration Instrument), daily Steps (pedometer).
House, et al. [47]	BrightArm Duo Rehabilitation System	Breakout 3D, Card Island, Remember that Card, Musical Drums, Xylophone, Pick & Place, Arm Slalom, Avalanche and Treasure Hunt.	2 sessions/week, progressed from 20 to 50 min per session. 8 weeks duration. Table tilt progressively increased each session from 0° to 20°.	None	Occupational Therapist	*Feasibility/Acceptability*: Acceptability and adherence (Custom questionnaire). *Function*: Arm movements and hand grasp repetitions (BrightArm system), UE ROM (mechanical goniometer (*Pre- to post-training*: Affected shoulder internal rotation (*p =* 0.05). *Pre-training to follow-up*: Affected shoulder adduction (*p =* 0.05), external rotation (*p =* 0.04), internal rotation (*p =* 0.01), affected elbow pronation (*p =* 0.03) and unaffected shoulder adduction (*p =* 0.04)), UE function (FMA, CAHAI-9, UEFI-20 (*Post-training to follow-up p =* 0.004), JHFT). *Strength*: Grasp strength (Hand grip, two finger pinch, three finger pinch), shoulder strength (wrist weights *(Pre- to post-training*: Anterior and lateral deltoid affected arm strength (*p =* 0.05). *Post-training to follow-up*: Lateral deltoid unaffected arm strength (*p =* 0.03)). *Emotions*: (BDI-II, NAB, HVLT-R, BVMT-R, TMT (*Pre- to post-training*: BDI-II (*p =* 0.04), BVMT-R (*p =* 0.007))). Pain: (NRS).
Jahn, et al. [26]	Nintendo Wii	Wii Sports, Family Trainer, Sports Island and Family Ski & Snowboard.	≥5 sessions/week, ≥30 min per session. 1 week duration.	None	Research Staff	*Feasibility/Acceptability*: Applicability and acceptability (Semi-structured interview). *Emotions*: Enjoyment and emotions (Semi-structured interview)
Sajid, et al. [46]	Nintendo Wii	Wii Fit (Game(s) not specified).	Similar mode, intensity and duration as EXCAP with balance addition. 12 week duration.	1) *EXCAP* ≥5 sessions/week. Walking: 60–70% HR reserve and RPE of 3–5. Increase daily steps by 5–20%. Resistance Bands: RPE of 3–5. Increase intensity by switching to higher resistance band. 2) *Usual Care.*	Exercise Physiologist	*Balance*: (SPPB* (Wii (*p =* 0.015), EXCAP v usual care (*p =* 0.038))). *Function*: (SPPB), *Physical Activity*: Steps per day (pedometer (EXCAP v usual care (*p =* 0.006)), 6MWT. *Strength*: Muscle strength and muscle mass (SPPB, hand grip, chest press, DEXA body composition (Wii lean muscle atrophy (*p =* 0.045)),
Tsuda, et al. [48]	Nintendo Wii	Wii Fit with Balance Board; Hula Hoop and Basic Step.	5 session/week, ≈20 min per session. Until hospital discharge. Median length of follow-up of 23.5 days.	None	Physiotherapist	*Feasibility/Acceptability*: Adherence (Adherence %), safety (CTCAE version 3.0). *Balance*: (One-leg stand time). *Function*: (Barthel Index, TUG, IADL). *Strength*: (Grip strength, knee-extension strength). *Emotions*: Psychosocial functioning (HADS).
Yoon, et al. [20]	IREX System	Birds & Balls, Conveyor, Drums, Juggler, Coconuts and Soccer.	3 sessions/week, 30 min per session, alongside COT 2 sessions/week, 30 min per session. 3 weeks duration.	1) *COT*. 5 sessions/week, 30 min per session. 3 weeks duration.	Occupational Therapist	*Function*: UE function (Modified Ashworth scale, MFT (Intervention (*p <* 0.001), intervention v control in SEF (*p =* 0.007). Control v intervention in hand function (*p =* 0.01)), BBT (Intervention (*p <* 0.001), intervention v control (*p =* 0.044)), FMA (Intervention total score (*p =* 0.0014) and SEF section (*p =* 0.048). Intervention v control during SEF (*p =* 0.012). Control v intervention during hand function (*p =* 0.046))), activities of daily living (K-MBI (Intervention (*p =* 0.005))).

*COP* Centre of pressure, *BFI* Brief Fatigue Inventory, *PSEFSM* Perceived Self-efficacy for Fatigue Self-management, *UE* Upper extremity, *ROM* Range of motion, *FMA* Fugl-Meyer Assessment, *CAHAI-9* Chedokee Arm and Hand Activity Inventory-9, *UEFI-20* Upper Extremity Function Index 20, *JHFT* Jebsen Hand Function Test, *BDI-II* Beck Depression Inventory, Second Edition, *NAB* Neuropsychological Assessment Battery, *HVLT-R* Hopkins Verbal Learning Test, Revised, *BVMT-R* Brief Visuospatial Memory Test, Revised, *TMT* Trail Making Test, *NRS* Numeric Pain Rating Sale, *EXCAP* Home-based walking and resistance intervention, *HR* Heart rate, *RPE* Rating of perceived exertion, *SPPB* Short Physical Performance Battery, *DEXA* Dual-energy x-ray absorptiometry, *6MWT* 6-min walk test, *CTCAE* Common Terminology Criteria for Adverse Events, *TUG* Timed up and go test, *IADL* Instrumental Activities of Daily Living, *HADS* Hospital Anxiety and Depression Scale, *IREX* Interactive Rehabilitation and Exercise, *COT* Conventional occupational therapy, *MFT* Manual Function test, *SEF* Shoulder/elbow/forearm, *BBT* Box and Block test, *K-MBI* Korean version of Modified Barthel Index

*Results of the SPPB weren’t segregated into the three separate categories

The interventions were delivered at various times, with one [44, 45] collecting baseline measures prior to surgery, post-surgery, and each week for 16 weeks. Two studies were implemented on patients with a current cancer diagnosis receiving treatment [26, 48], with one study taking place 9.5 years post-surgery [47]. The study by Sajid, et al. [46] took place 62 month after androgen deprivation therapy (ADT) initiation, whilst Yoon, et al. [20] assessed participants nine month after diagnosis. Three interventions were carried out within a hospital setting [26, 48, 49], two in the participants’ home [44–46] and one in a clinical laboratory [47] (Table 4). The interventions were delivered by a physiotherapist [48, 49], an occupational therapist [20, 47], nurse [44, 45], research staff [26] or an exercise physiologist [46] (Table 5).

### Feasibility and acceptability of an exergaming intervention

Feasibility of exergaming interventions was assessed through retention rates and adherence rates whilst acceptability was assessed through custom acceptability questionnaires and interviews.

Five studies assessed the feasibility and acceptability of using exergaming for cancer rehabilitation, determined through adherence percentage [44, 45, 47, 48], retention rate [44, 45, 48], a custom evaluation questionnaire [44, 45, 47, 49] and a semi-structured interview [26].

The study by Hoffman, et al. [44, 45] reported an adherence rate of 96.6% after six weeks, which fell to 87.6% after 16 weeks. All participants within this study continued throughout the 16 weeks, giving a retention rate of 100%. The participants in the study by Tsuda, et al. [48] had an adherence rate of 62% and a retention rate of 56%; however, only one dropout wasn’t due to ill health. Any sessions missed by those in the study by House, et al. [47] were made up during alternative days, leading to a 100% adherence rate. Of the 12 participants enrolled, only six completed the testing, leading to a 50% retention rate.

Only one study assessed the safety of the exergaming intervention using a set of criteria to grade any adverse effects. Tsuda, et al. [48] found there to be no musculoskeletal or adverse events associated with the intervention.

The studies that implemented a custom evaluation questionnaire showed high acceptability of 5.8/6 [44, 45] and 3.9/5 after four weeks increasing to 4.5/5 after eight weeks, with higher scores indicative of a high level of acceptance [47]. The questionnaire used by Betker, et al. [49] only included three questions; however, one of these showed that the exergaming intervention was preferred to exercise programs the participant had previously partaken in. When participants were asked for their opinion of the exergaming interventions, they were described as fun, easy to use, convenient, comfortable, relaxing and would recommend their use [26, 44, 45, 47].

### The outcomes of exergaming interventions

Results of the exergaming interventions were assessed through many measures. These included balance, function, physical activity, strength, fatigue, emotions, cognition and pain.

### Physical functioning

Betker, et al. [49] assessed pre- and post-intervention balance and falls during numerous 20 s tests, such as eyes open, eyes closed, unipedal, bipedal and differing surfaces, along with COP measurements. The sole participant in this study improved their fall count (unable to maintain balance) from 10 falls from 12 tests pre-intervention to one fall from the same tests post-intervention, with an improved COP range during all tests.

One study assessed participants’ perceived balance confidence, using the Activities-Specific Balance Confidence Scale [44, 45]. Pre-surgery scores were 86%, falling to 72.8% post-surgery. At post-intervention, the perceived balance confidence scores increased to 83.7% and then to 88.9% after week six and 16, respectively. The average length of hospital stay was eight days, with the intervention beginning on average 32 h following discharge.

The Short Physical Performance Battery (SPPB) was used in one study [46], which includes a balance component. The results of this, however, weren’t segregated from the other components of this test. The final study to assess balance used a baseline and post-exercise one leg standing time [48], finding no significant improvement (left leg *p* = 1; right leg *p* = 0.1).

House, et al. [47] assessed function through arm movements, hand grasp repetitions, upper extremity (UE) ROM, Fugl-Meyer Assessment (FMA), Chedokee Arm and Hand Activity Inventory-9 (CAHAI-9), Upper Extremity Function Index 20 (UEFI-20) and Jebsen Hand Function Test (JHFT). Arm movements and hand grasp repetitions steadily increased from session one through to 16. ROM increased among almost all movements assessed, with only a decrease in unaffected shoulder external rotation. Significant improvements were seen in the affected shoulder internal rotation (*p =* 0.05) between pre- and post-training, the affected shoulder adduction (*p =* 0.05), internal rotation (*p =* 0.01) and external rotation (*p =* 0.04) and the affected elbow pronation (*p =* 0.03) between pre-training and the eight week follow-up post-intervention. Improvements were also seen in the other aforementioned tests, barring the JHFT in the unaffected shoulder which decreased. UEFI-20 was significantly greater at the eight week follow up compared to pre-training (*p =* 0.004).

Function was partly measured through the implementation of the SPPB in the study by Sajid, et al. [46] which includes the 4-m gait speed test. Again, these results weren’t isolated from the other components of this test. The Barthel Index, Timed Up and Go (TUG) test and the Instrumental Activities of Daily Living scale (IADL) were used in another study [48]. No difference was seen between baseline and post-exercise measures for the Barthel Index; however, the TUG showed a decrease (*p* = 0.58), with slight improvements seen during the IADL (*p* = 1).

The Modified Ashworth scale, Manual Function Test (MFT), Box and Block Test (BBT), FMA and the Korean version of the Modified Barthel Index (K-MBI) was used in the study by Yoon, et al. [20]. Significant improvements in functional dexterity were found through the BBT (*p =* 0.044) in the combined exergaming and COT group compared to the COT group alone. It was also found that shoulder/elbow/forearm (SEF) function improved significantly among the combined exergaming group during the MFT (*p =* 0.007) and FMA (*p =* 0.012) in comparison to the COT group. Hand function improved significantly within the control group in comparison to the combined exergaming and COP group (*p =* 0.01, *p =* 0.046) during the MFT and FMA, respectively.

A pedometer was used to assess daily steps completed, whilst the Self-efficacy for Walking Duration Instrument was used to assess perceived walking self-efficacy among participants in the study by Hoffman, et al. [44, 45]. Steps per day increased from 4650 during week one, to 6393 during week six, before fluctuating on a weekly basis thereafter. Perceived self-efficacy for walking duration was based on participant’s perception of being able to walk for 30 min. This self-efficacy dropped from 96.4% pre-surgery to 47.4% post-surgery, before increasing to 99.4% at the end of the intervention at 16 weeks.

A pedometer was also used to measure steps taken per day in another study [46]. The results showed an increased in all three groups, with the exercise group significantly greater than the usual care group (*p =* 0.006).

House, et al. [47] assessed UE strength through a hand grip test, a two and a three finger pinch test as well as wrist weights used to measure shoulder strength, all of which improved in the affected shoulder from pre- to post-training and again after the eight week follow-up. Anterior and lateral deltoid strength significantly improved between pre- and post-training (*p =* 0.05) following the intervention. A similar trend was also seen for the unaffected arm strength, despite the two fingers pinch score decreasing. Lateral deltoid strength of the unaffected shoulder increased significantly between pre-training and eight week follow-up (*p =* 0.03).

Chest press, handgrip dynamometer test and a dual energy x-ray absorptiometry (DEXA) scan was completed to assess muscle mass and muscle strength in another study [46]. Handgrip scores and lean muscle mass (*p =* 0.045) decreased in the Wii group, whilst the EXCAP group seen an increase in handgrip strength, with a decrease in lean muscle mass, both of which non-significant.

The final study to assess strength [48] used grip strength and knee extension strength. Knee extension strength increased non-significantly post-intervention, in comparison to baseline measures. Grip strength increased in the left hand, with a decrease in the right hand, both of which non-significant.

### Symptoms

Only one intervention [44, 45] assessed fatigue. This was done using the Brief Fatigue Inventory (BFI) and also assessed participants’ perceived self-efficacy for managing fatigue using the Perceived Self-efficacy for Fatigue Self-management (PSEFSM) instrument. On a scale of 0–10, with 10 being ‘most severe’, the scores of the BFI reveal a pre-surgery score of 3.3, rising to 4.8 post-surgery. This value fell to 2.8 after week six, and again to 1.32 at week 16. Perceived self-efficacy for managing fatigue rose from 7.0 pre-surgery to 7.1 post-surgery before falling until week two at 5.4, before rising to 7.7 and remaining between 7 and 9 for the remainder of the 16 week intervention.

The participant in the study by Betker, et al. [49] said they had fun partaking in the exergaming intervention. They stated they experienced a loss of time awareness, as found through a short custom questionnaire.

The study by House, et al. [47] used the Beck Depression Inventory second edition (BDI-II), the Neuropsychological Assessment Battery (NAB), Hopkins Verbal Learning Test, Revised (HVLT-R), the Brief Visuospatial Memory Test, Revised (BVMT-R) and the Trail Making Test (TMT) A and B. Improvements were seen between pre- and post-training in all tests, with the BDI-II and BVMT-R improving significantly (*p =* 0.04 and *p =* 0.007, respectively). No change was seen in the ‘Person’ category in the NAB, and the TMT-A showed a non-significant decrease. No significant improvements were seen between pre-training and at eight week follow-up, despite improvements in eight out of 12 tests. Tsuda, et al. [48] used the Hospital Anxiety and Depression Scale (HADS) to measure the psychosocial functioning of participants. Both anxiety and depression decreased within this study, with anxiety scores approaching significance (*p =* 0.055).

Interviews were carried out in the study by Jahn, et al. [26]. Through these interviews, participants responded saying they felt a decrease in negative emotions such as stress and that they felt more relaxed and had an increased mood state following the intervention.

Pain was only assessed in one study, using the Numeric Pain Rating Scale (NRS) [47]. Pain showed a slight decrease, with the worst pain reported in week one as 5/10, with a decrease of 1.1 over the eight week period (*p* = 0.1).

## Discussion

This is the first systematic review to synthesise current evidence in order to describe exergaming interventions delivered to individuals with a current or previous diagnosis of cancer and to explore the feasibility, acceptability and outcomes of such interventions. The interventions found through this review showed great variability to one another.

Due to the variability of the interventions included, it is difficult to conclude which method of delivery would prove most advantageous. No one mode of intervention appeared to be most beneficial, although the majority used the Nintendo Wii. Almost half of the interventions were delivered within the hospital, with two delivered at home, indicating the suitability of exergaming interventions within both environments. Interventions showing significant improvements in health outcomes were delivered by an occupational therapist. The only other intervention to report an improvement was delivered by an exercise physiologist. It would appear exergaming interventions are most successful when delivered by a qualified healthcare professional or a professional with knowledge of exercise prescription. Duration and frequency of interventions varied greatly, however longer and more frequent interventions didn’t incur greater improvements. Balance, function, emotions and fatigue were the health outcomes most frequently assessed and targeted by exergaming interventions.

Exergaming interventions appear to be feasible and acceptable to individuals with cancer. It is difficult, however, to draw firm conclusions regarding its benefits due to the heterogeneity in the delivery of interventions and the outcome measures assessed within the included studies.

Adherence to interventions delivered to individuals with cancer and consisting of exercise, is relatively low [16, 18, 21] potentially due to the monotonous nature of aerobic and resistance training [50]. Numerous other factors have been shown to be associated with adherence rates among cancer patients, including, but not limited to, exercise history, fatigue levels, body mass index and level of education [51, 52]. Adherence percentages within this review [44, 45, 47, 48] were positive, with the lowest being 62%; however only one drop-out in this study wasn’t due to ill health, displaying high feasibility among those who are well, but also highlighting a challenge of delivering exercise interventions to a vulnerable group of individuals. Only one study within this review assessed the safety of the intervention using a set of criteria to grade any adverse events. No musculoskeletal or adverse events occurred [48].

Exergaming interventions have been shown to be acceptable to older adults [53], and those with chronic conditions, including PD [24], MS [54], chronic obstructive pulmonary disease (COPD) [55] and stroke [56] patients, concurring with results from the current review. Older adults [53], PD [24] and post-stroke patients [56] found the interventions to be fun and enjoyable, whilst older adults [53], PD [24] and MS patients [54] were motivated to participate. MS patients [54] showed greater intrinsic motivation through the Flow State Scale than those undertaking traditional training. Post-stroke patients [56] also found the exergaming intervention to be useful, if not better, than conventional rehabilitation, and would recommend its use to other patients. Conversely, COPD patients [55] accepted a pulmonary rehabilitation program (PRP) more than the exergaming intervention, albeit non-significantly. Barry, et al. [24] found that fast-paced and complex games caused difficulties and that games should be tailored according to the needs of the clinical population.

Exergaming has already been shown to be an effective method to increase physical activity in chronic disease populations, including PD [24], MS [31], cystic fibrosis [32] and those who have recently suffered a stroke [33], with little research assessing exergaming in a cancer population. Through this review, exergaming appears to improve several health aspects, such as balance, function, physical activity, strength, fatigue, emotions, cognition and pain among cancer patients.

Balance was shown to improve through two interventions within this review [44, 45, 49]. Fall counts and postural sway decreased in one study [49], whilst Hoffman, et al. [44, 45] found that participants’ balance self-efficacy increased. Exergaming has also been shown to improve balance among older adults [27, 53] and clinical populations, including PD [24], MS [54, 57] and post-stroke [33] patients. A study among older participants found a Wii intervention to significantly improve results of the Berg Balance Scale, between baseline and week four compared to standard care alone [27].

Function was assessed in four interventions [20, 46–48] within this review, and was shown to improve, whether this was improved arm movements, hand grasp repetitions or functional dexterity. Exergaming interventions have been shown to have mixed results on functionality among older adults and clinical populations. Among older participants, dual-task function has been shown to improve in some studies, following a systematic review; however, many significant findings weren’t in comparison to a control group [34]. PD patients showed an increase in functionality following a variety in exergaming intervention, through a variety of tests as shown through a systematic review [24], whilst MS patients displayed mixed results, with non-significant improvements in the TUG and 25 ft walk test, and significant improvements for both the exergaming and control group during the Dynamic Gait Index [31]. PD patients’ function has shown to improve, which coincides with the findings from the current review. MS and older participants’ function meanwhile is not as conclusive.

Physical activity was assessed within two interventions in this review [44–46]. Steps per day were shown to increase among those who partook in an exergaming intervention within this review; however, not as much as a standard exercise group. Although little research exists in exergaming interventions, standard exercise interventions have shown improvements in exercise capacity among COPD patients, potentially allowing for an improvement in physical activity [58, 59].

Exergaming interventions were shown to improve several measures of strength, assessed during three interventions [46–48], throughout this review. One study found that despite improvements, a standard exercise group improved more than the exergaming group [46]. PD patients have shown significant improvements in their strength following an exergaming intervention [60]. These improvements were seen for both the exercise and exergaming group within the study, with no significant difference between the two. Post-stroke patients saw a significant improvement of strength following an exergaming intervention, whilst recreation therapy showed a non-significant increase [61]. This previous literature coincides with the findings from the current review. Despite improvements, it is inconclusive as to whether exergaming is more beneficial than a standard exercise intervention.

Exergaming was seen to decrease levels of fatigue in the one intervention [44, 45] where it was assessed through the current review. This finding concurs with findings of exergaming interventions among PD [62], MS [63] and COPD [64] patients, along with those who were undergoing a PRP [65].

Four interventions [26, 47–49] in this systematic review found that exergaming had a positive impact on the emotions and cognition of cancer patients. Depression, mood and relaxation all improved following the interventions. Older participants have shown an increase in emotions and cognition following an exergaming intervention. A scoping review carried out by Klompstra, et al. [53] found that three studies reported an increase in cognitive function [66–68], with one showing a decrease in depressive symptoms [68]. Likewise, PD [69] patients have also shown to have an improved mood, whilst COPD [55] patients have shown a significant decrease in depressive symptoms and a non-significant decrease in anxiety among the exergaming group. Changes were however less than the PRP alone, which seen significant decreases among both outcomes. The findings of the current review coincide with this previous literature.

Pain was only assessed in one intervention in this review [47], showing a slight decrease following an exergaming intervention. These findings are in keeping with other studies which have implemented exergaming, conducted in post-stroke patients [56], those having undergone a knee replacement [70] and also with UE dysfunction [71]. A systematic review also revealed that four of seven studies found significant improvements in musculoskeletal pain following exergaming interventions [72].

This systematic review is not without its limitations. The studies which were included within this review were vastly different from one another in terms of the console used, game played, the diagnosis of cancer and the outcomes assessed, which made analyses of the effects of exergaming difficult to derive, and a meta-analyses unable to be performed. Therefore no conclusion could be made on a single exergame or specific console, rather exergaming generically as a whole. Due to the relatively small number of studies included, conclusions drawn are tentative, however this review offers important insights regarding the delivery of exergaming to individuals with cancer. This review only found one study which assessed the safety of the intervention which is an important factor in determining feasibility. The safety of interventions should be considered in future studies applying exergaming interventions in a cancer population by reporting adverse events using set criteria. Other threats to internal and external validity exist within the included studies. Only one of the studies included was a randomised control trial thus conclusions on the outcomes of exergaming are taken with caution. Reviews with few or no randomised controlled trials are still valuable for inclusion within systematic reviews as they provide information regarding the feasibility of an intervention [73]. They can also prove to be of high value and of high interest to patients, healthcare professionals and stakeholders [74, 75]. The sample sizes within the studies were also relatively small, in one case with only one eligible participant. Due to this, it is difficult to draw firm conclusions from the results of the analyses due to the limited statistical power. The criteria of any cancer patients meant that a variety of cancers were researched, meaning no conclusion could be made on a specific diagnosis or disease severity. The lack of control group in many of the studies meant that it was difficult to determine whether the exergaming interventions were the cause of improvement, or whether these would have been shown following a standard exercise based regime. The inclusion criterion for this review was very broad which allowed for a range of studies to be included. In doing so, it provides a general insight into how exergaming has been used for patients with a current or previous diagnosis of cancer.

It is clear, through this review, that due to the scarcity of literature within this field, more research is required. Future research should explore common outcome variables during exergaming interventions, suitable to all cancer groups. It is also recommended that a control group, using standard exercise therapy, should be included in order to determine whether improvements are solely due to the exergaming intervention. Studies should also look to implement longer follow-ups in order to assess the feasibility, acceptability and outcomes of an exergaming intervention over a prolonged period of time.

## Conclusion

In conclusion, the content of the interventions delivered to cancer patients show great heterogeneity, along with a wide variety of health outcomes assessed, therefore it is difficult to conclude its effectiveness. However, adherence rates and enjoyment appear greater during exergaming interventions compared to standard care, highlighting the feasibility and acceptability of this type of intervention delivery for adults with cancer.

## Appendix

Search Strategy.

(exergam* or activ* n3 video n3 gam* or activ* n3 videogam* or human n3 computer n3 interaction or virtual n3 reality or virtual n3 world or augment* n3 reality or mobile n3 app* or exert* n3 interfac* or electronic n3 gam* or activ* n3 gam* n3 play or virtual n3 rehab* or augment* n3 rehab* or mHealth or Wii or Eye n3 Toy or Eyetoy or Kinect or DDR or Dance n3 Dance n3 Revolution or IREX) and (neoplasm* or cancer* or carcino* or leukemia or leukaemia or tumor or tumour or NSCLC or non n3 small n3 cell or thorac* or postthorac*).

## Abbreviations

6MWT: 6-minutemin walk test
ADT: Androgen deprivation therapy
BBT: Box and Block Test
BDI-II: Beck Depression Inventory second edition
BFI: Brief Fatigue Inventory
BVMT-R: Brief Visuospatial Memory Test, Revised
CAHAI-9: Chedokee Arm and Hand Activity Inventory-9
CASP: Critical Appraisal Skills Program
COP: Centre of pressure
COPD: Chronic obstructive pulmonary disease
COT: Conventional occupational therapy
CRF: Cancer-related fatigue
CTCAE: Common Terminology Criteria for Adverse Events
DEXA: Dual energy x-ray absorptiometry
FMA: Fugl-Meyer Assessment
HADS: Hospital Anxiety and Depression Scale
HR: Heart rate
HVLT-R: Hopkins Verbal Learning Test, Revised
IADL: Instrumental Activities of Daily Living
IREX: Interactive Rehabilitation and Exercise
JHFT: Jebsen Hand Function Test
K-MBI: Korean version of the Modified Barthel Index
MFT: Manual Function Test
MS: Multiple sclerosis
NAB: Neuropsychological Assessment Battery
NRS: Numeric Pain Rating Scale
PD: Parkinson’s disease
PRP: Pulmonary rehabilitation program
PSEFSM: Perceived Self-efficacy for Fatigue Self-management
QOL: Quality of life
ROM: Range of motion
RPE: Rate of perceived exertion
SEF: Shoulder/elbow/forearm
SPPB: Short Physical Performance Battery
TMT: Trail Making Test
TUG: Timed Up and Go
UE: Upper extremity
UEFI-20: Upper Extremity Function Index 20
